# A Note of Caution: Gramicidin Affects Signaling Pathways Independently of Its Effects on Plasma Membrane Conductance

**DOI:** 10.1155/2021/2641068

**Published:** 2021-10-21

**Authors:** Frances Evans, Julio A. Hernández, Federico Cabo, Silvia Chifflet

**Affiliations:** ^1^Departamento de Histología y Embriología, Facultad de Medicina, Universidad de la República, Gral. Flores 2125, 11800 Montevideo, Uruguay; ^2^Sección Biofísica, Facultad de Ciencias, Universidad de la República, Iguá s/n esq. Mataojo, 11400 Montevideo, Uruguay; ^3^Departamento de Bioquímica, Facultad de Medicina, Universidad de la República, Gral. Flores 2125, 11800 Montevideo, Uruguay

## Abstract

Gramicidin is a thoroughly studied cation ionophore widely used to experimentally manipulate the plasma membrane potential (PMP). In addition, it has been established that the drug, due to its hydrophobic nature, is capable of affecting the organization of membrane lipids. We have previously shown that modifications in the plasma membrane potential of epithelial cells in culture determine reorganizations of the cytoskeleton. To elucidate the molecular mechanisms involved, we explored the effects of PMP depolarization on some putative signaling intermediates. In the course of these studies, we came across some results that could not be interpreted in terms of the properties of gramicidin as an ionic channel. The purpose of the present work is to communicate these results and, in general, to draw attention to the fact that gramicidin effects can be misleadingly attributed to its ionic or electrical properties. In addition, this work also contributes with some novel findings of the modifications provoked on the signaling intermediates by PMP depolarization and hyperpolarization.

## 1. Introduction

Ever since its discovery in the late thirties [1], gramicidin has been first employed as an antibiotic [2] and later as an experimental model for the study of the basic properties of ionic channels [3]. Natural gramicidin produced by *Bacillus brevis* is a mixture of several molecules, predominantly gramicidin A [3]. Gramicidins are exclusively selective for monovalent cations, with a predominance of potassium over sodium (ibid). As a consequence of its properties as an ionic channel, the ionophore has also been widely employed as an additional experimental tool to provoke modifications in the plasma membrane potential in diverse cellular systems, mainly as a depolarizing agent [4–10]. The effect of gramicidin on the plasma membrane potential (PMP) depends on the extracellular ionic composition [11–13]. Under normal resting conditions, where the PMP is close to the equilibrium potential of potassium, gramicidin determines depolarization by mediating sodium entry. If extracellular sodium is replaced by a nonpermeant cation (e.g., choline), gramicidin provokes hyperpolarization. It has also been known for a long time that, besides its properties as an ionophore, gramicidin affects the membrane structural organization by direct interaction with constitutive lipids [14, 15]. In addition, gramicidin at high concentrations has been shown to directly inhibit purified Na, K-ATPase [16]. In recent years, the possibility of utilizing gramicidin and its derivatives as potential therapeutic agents for several diseases has reactivated the interest in this molecule [17–19].

In previous work, we showed that modifications in the PMP of diverse epithelial cells in culture provoke characteristic reorganizations of the cytoskeleton and intercellular junctions. Thus, membrane depolarization determines a reduction in the actin peripheral ring and relocalization of F-actin [5, 20], whereas plasma membrane hyperpolarization reinforces circumferential actin and increases junction stability [21]. To unearth the basic molecular mechanisms underlying the cytoskeletal responses to unspecific plasma membrane depolarization, we explored its effects on some putative signaling intermediates that could potentially be involved [22]. In the course of these studies, we came across some results that could not be interpreted in terms of the properties of gramicidin as an ionic channel in the plasma membrane. The purpose of the present work is to communicate these results and, in general, to draw attention to the fact that some gramicidin effects can be misleadingly attributed to its ionic or electrical properties.

## 2. Materials and Methods

This study uses methods previously employed by us [5, 20–22], and their description below partly reproduces their wording.

### 2.1. Reagents and Solutions

Chemicals and drugs were from Sigma (Munich, Germany) unless otherwise specified. Control solution (CS): 137 mM NaCl, 5.4 mM KCl, 1.02 mM NaH_2_PO_4_, 3.6 mM CaCl_2_, 0.8 mM MgSO_4_, 10 mM HEPES pH 7.4. Potassium gluconate (KGln), sodium gluconate (NaGln), choline chloride (Cho), and N-methyl-D-glucamine chloride (NMDG) solutions as CS, but replacing NaCl with equimolar potassium gluconate, sodium gluconate, choline chloride, or NMDG, respectively, and Na_2_SO_4_ solution as CS, but replacing NaCl with equiosmolar Na_2_SO_4_. Gramicidin A and valinomycin were prepared as 1000x stock solutions (1 mg/ml) in dimethyl sulfoxide (DMSO) and kept at -20°C until use. For the experiments, gramicidin was diluted to 1 *μ*g/ml in CS (Gra) or in Cho (Gra-Cho). Analogously, valinomycin was diluted to 1 *μ*g/ml in CS (Val). DMSO alone (0.1% vol/vol) did not affect cell viability or any of the properties studied in this work.

### 2.2. Cell Culture

Fresh bovine eyes were kindly provided by a certified slaughterhouse (see Acknowledgments). Corneal endothelial (BCE) cells were obtained and cultured as described previously [5]. In brief, after two washes with 70% ethanol, the corneas were dissected in the laminar flow chamber and then treated with trypsin (0.25%)-EDTA (0.02%) in Ca^2+^- and Mg^2+^-free PBS for 20–30 min. The endothelial cells of each cornea were carefully scraped with a blunt spatula and placed in a 35 mm tissue culture plate containing MEM supplemented with 10% serum, 50 *μ*g/ml gentamicin, 0.25 *μ*g/ml amphotericin B, and 50 *μ*g of total protein/ml of retinal extract and kept in a humidified tissue culture incubator at 37°C in 5% CO_2_. Once confluence was reached (usually 7–10 days), the primary cultures were subcultured in the same medium and grown either on glass coverslips or on 60 mm tissue culture plates. For this study, we utilized cells from the 1st to the 5th passages that had achieved confluence at least 5 days before the experiments.

### 2.3. Modifications of the Plasma Membrane Potential

Depolarizations and hyperpolarizations of the PMP in BCE cells were achieved as in previous works from our laboratory (see above). Briefly, cells were incubated for 30 min at room temperature in CS or in depolarizing (KGln, NaGln, and Na_2_SO_4_ solutions or Gra) or hyperpolarizing (Cho, NMDG, and Val or Gra-Cho) conditions. In the above mentioned studies, we have demonstrated the effect on the PMP of the corresponding solutions in BCE cells.

### 2.4. Fluorescence Microscopy

Unless otherwise specified, the following procedures were performed at room temperature. Confluent cells grown on glass coverslips were treated as in Section 2.3 and fixed in 4% paraformaldehyde in Dulbecco's PBS (CaCl_2_ and MgCl_2_ added immediately before use) for 15 min. The cells were then washed with PBS and permeabilized with 0.1% Triton X-100 for 5 min for phospho-ERK 1/2 (pERK) and phosphotyrosine (pTyr) and for 15 min for diphosphorylated myosin light chain (ppMLC). After this, the cells were washed three times with PBS and incubated with an anti-pERK antibody (cat# M7802, RRID: AB_260658; Sigma) at a 1/500 dilution, an anti-ppMLC antibody (T18/S19; cat# 3674; RRID: AB_2147464; Cell Signaling Technology, Beverly, MA) at a 1/200 dilution, or an anti-pTyr antibody (cat# P3300, RRID:AB_477335, Sigma) at 1/100 dilution. After incubation with the primary antibody, the cells were washed three times with PBS and incubated with an anti-mouse Alexa Fluor 594-conjugated secondary antibody (cat# A-11005, RRID: AB_141372; Molecular Probes Inc.) for pERK and pTyr or an anti-rabbit Alexa Fluor 594-conjugated secondary antibody (cat# A-21207; RRID: AB_141637; Thermo Fisher Scientific, San Jose, CA) for ppMLC. All the primary and secondary antibodies were diluted in 1% BSA/PBS and incubated for 60 min at 37°C in a humid chamber. For actin and nuclear visualization, 2 *μ*g/ml fluorescein isothiocyanate-phalloidin and 50 *μ*M Hoechst 33258, respectively, were added to the secondary antibody solution. For microscopy, the cells were washed three times with PBS and the coverslips mounted in 5 : 1 glycerol : TRIS (1.5 M, pH 8.8). The monolayers grown on glass coverslips were viewed with a Nikon 50i epifluorescence microscope (Nikon, Tokyo, Japan) and photographed with a Moticam Pro 282B camera and Moticam Image Advanced 3.2. software (Motic, Xiamen, China).

To estimate the extent of nuclear localization of pERK, we employed the Hoechst-stained nuclear images to make nuclear masks utilizing the Magic Wand Tool of the Adobe Photoshop CS5 Extended software (RRID: SCR_014199, Adobe Systems, Mountain View, Calif., USA). The masks were then overlapped to the corresponding pERK-stained images and utilized to obtain the integrated density (ID) using the measurement command of the same software. The number of pERK-positive nuclei was then determined for each condition. We considered a nuclear image to be positive when its ID was at least 3 times larger than the mean ID of the background. A similar procedure was utilized to compare the relative extents of ppMLC fluorescence between the different experimental conditions. The ID values were used to make a histogram plot. The ID value of each dot was normalized with respect to the higher value of ID among the different conditions of the same experiment. For every experiment, the IDs of at least 10 images per condition were quantified.

### 2.5. Western Blots

After treatment, cells were processed as follows. For pERK, they were scraped in the following ice-cold lysis buffer: 100 mM NaCl, 30 mM HEPES pH 7.5, 20 mM NaF, 1 mM EGTA, 1% Triton X-100, 1 mM Na_3_VO_4_, 1 mM phenylmethanesulfonyl fluoride (PMSF), and an antiprotease cocktail (antipain, pepstatin A, and leupeptin; final dilution 10 *μ*g/ml of each; [23]), added at the moment of use. Then, the appropriate amount of a 4x Laemmli buffer was added to each sample. For ppMLC, the cells were lysed in Laemmli buffer. For every treatment, the samples were afterwards boiled for 5 min and then run on a 4% stacking-10 or 12% running sodium dodecyl sulfate- (SDS-) polyacrylamide gel. To analyze ppMLC, 200 *μ*g of protein was loaded into each well, whereas 100 *μ*g was loaded for pERK. Next, proteins were transferred to a PVDF membrane (Amersham-GE, Healthcare) in protein transfer buffer (25 mM Tris, 192 mM glycine, 20% methanol, pH 8.3). Nonspecific binding sites of the membrane were blocked with 5% fat-free milk in PBS for 30 min at 37°C and incubated overnight at 4°C with the same anti-ppMLC or anti-pERK antibodies used for immunofluorescence, both at 1 : 500 dilution in 1% milk, 0.05% Tween 20 in PBS. After washing three times with 0.05% Tween 20-PBS, the membranes were incubated for 1 h at room temperature with the corresponding horseradish-peroxidase-coupled antibody (cat# A4416; RRID: AB_258167 or cat# A0545; RRID: AB_257896), diluted 1/10000 in the same solution as the primary antibodies. The blots were developed with a noncommercial enhanced chemiluminescence solution [24], exposed to film (Carestream Health, Inc., Rochester, NY), and scanned. The integrated density (ID) of each band was quantified with ImageJ (RRID: SCR_003070). Following [25], transfer controls and normalizations were performed by quantifying the ID of the Ponceau S-stained membranes with the same software. To compare data from different blots, the bands were normalized to the sum of all the bands belonging to one experiment (set equal to 100%).

### 2.6. Statistics

At least three independent experiments were performed per case. For fluorescence microscopy, each experiment was done in duplicate and at least 5 images selected at random were captured per coverslip. For each quantitative determination, all of the 10 images were utilized. Results are expressed as means ± SD. Statistical significance was determined by one-way analysis of variance (ANOVA) followed by post hoc Tukey's test for multiple comparison analysis, employing GraphPad Prism software (GraphPad Software, San Diego, CA). Statistical significance was considered *p* < 0.05.

## 3. Results

When BCE cells are incubated in physiological solutions containing gramicidin, they undergo PMP depolarization [5, 20]. On the contrary, in the absence of extracellular sodium, gramicidin provokes hyperpolarization [5]. These findings suggest that BCE cells possess a significantly larger permeability for potassium than for sodium, a typical property of most nonexcitable animal cells. We have made use of these biophysical characteristics of BCE and other epithelial cells in culture to explore the effects of modifications of the PMP on the cytoskeletal organization and on the stability of intercellular junctions [5, 20, 21]. Here, to put into evidence the nonionophoric effects of gramicidin, we made comparisons with other procedures that modify the PMP of BCE cells and study their consequences on some signaling intermediates. Namely, for the depolarizing solutions, we substituted extracellular NaCl by KGln, NaGln, or Na_2_SO_4_. To produce hyperpolarization, NaCl was replaced by Cho, NMDG, or Val (see Materials and Methods). Whereas Cho and NMDG are poorly permeable cations that produce hyperpolarization by substituting extracellular sodium, Val operates as an ionophore highly selective for potassium, thus improving the conductivity of the plasma membrane for this ion.

### 3.1. Effect of Gramicidin A on ERK Phosphorylation and Localization

**Figure 1:**
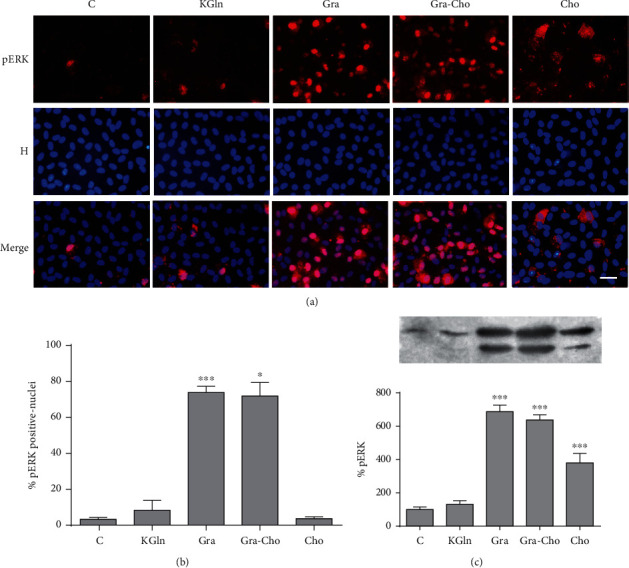
Effect of gramicidin A on ERK phosphorylation and localization in BCE cells. (a) Double-stained images of pERK and nuclei (H: Hoechst) for different conditions (C: control; KGln; Gra; Gra-Cho; Cho). (b) Plot of percentage of pERK positive nuclei. In agreement with (a), the percentage of pERK-positive nuclei is significantly larger for Gra and Gra-Cho compared to control and the other treatments (^∗^*p* < 0.05; ^∗∗∗^*p* < 0.0001). Although Cho determines a higher global immunofluorescent signal of pERK with respect to control (see (a)), its distribution is diffuse throughout the cytoplasm with a low nuclear component. (c) Western blots of pERK in cell lysates for all the conditions. Similarly to (a), the blots reveal an important increase in pERK in response to the gramicidin or gramicidin-choline treatments and a somewhat augmented signal for choline alone. KGln did not determine any modification. Asterisks indicate significant differences with respect to control (^∗∗∗^*p* < 0.0001). The signal for Cho is also significantly different with respect to Gra (*p* < 0.01) and Gra-Cho (*p* < 0.05) (not indicated in the figure). Load and transfer controls and quantifications were done as described in Section 2.5. Bar: 30 *μ*m.

**Figure 2:**
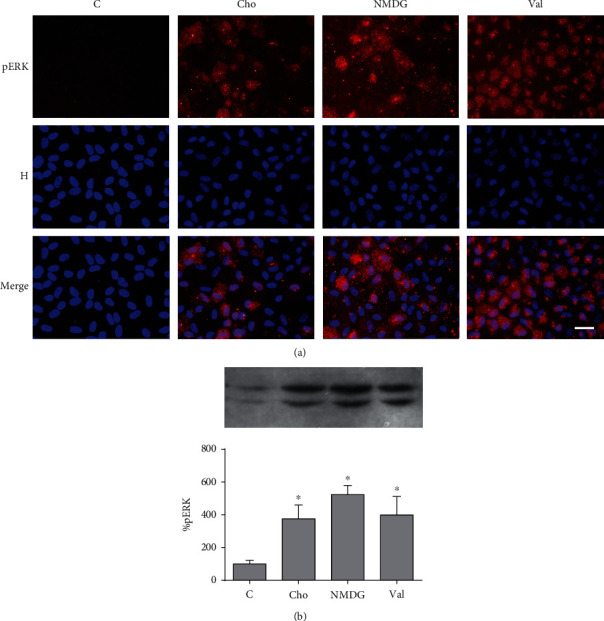
Effect of plasma membrane hyperpolarization on pERK in BCE cells. (a) Double-stained images of pERK and nuclei (H: Hoechst) for different conditions (C: control; Cho, NMDG, and Val). The hyperpolarizing treatments provoke a diffuse increase of the pERK signal throughout the cytoplasm. (b) Western blots of pERK in cell lysates for all the conditions. Similarly to (a), the blots reveal an important increase in pERK for all the hyperpolarizing treatments. Asterisks indicate a significant difference with respect to control (^∗^*p* < 0.05). Load and transfer controls and quantifications were done as described in Section 2.5. Bar: 30 *μ*m.

The extracellular signal-regulated kinase 1/2 (ERK1/2) associates with the cytoskeleton and regulates its organization [26].  Figure 1(a) shows that NaCl replacement by KGln (a depolarizing procedure [5]) did not elicit any change in the intensity or localization of the pERK (active form of ERK) signal in BCE cells, as previously reported by us for this and other depolarizing solutions (i.e., NaGln and Na_2_SO_4_) [22]. In contrast, the hyperpolarizing procedures (i.e., Cho (Figure 1(a)) and NMDG or Val (Figure 2(a)); see below) produced an increase in ERK phosphorylation. The experiments in Figure 2 were performed to exclude the possibility that Cho affects ERK via a mechanism independent of PMP hyperpolarization. As can be seen, the three hyperpolarizing procedures produced analogous effects on ERK. However, both gramicidin alone (a depolarizing agent when incorporated into the control solution, see above) or in combination with Cho (Gra-Cho, a hyperpolarizing solution in this system, as commented) determined a much larger increase in the number of pERK-positive cells (Figure 1(a)) with an intense nuclear signal (Figure 1(b)). As a difference with Gra, the increase in the pERK signal determined by the hyperpolarizing procedures is predominantly cytoplasmic (Figures 1(a) and 2(a)). In agreement with these results, Western blot analysis revealed that these two conditions determined a very intense increase in the pERK bands with respect to control or to other depolarizing and hyperpolarizing procedures (Figures 1(c) and 2(b)). Also, consistent with the immunofluorescence images, hyperpolarization alone (Cho) caused a noticeable increase in the corresponding band signals (Figure 1(c)). These results suggest that the increase in pERK promoted by gramicidin A does not depend on its effects as an ionophore of the plasma membrane.

### 3.2. Effect of Gramicidin A on ppMLC

**Figure 3:**
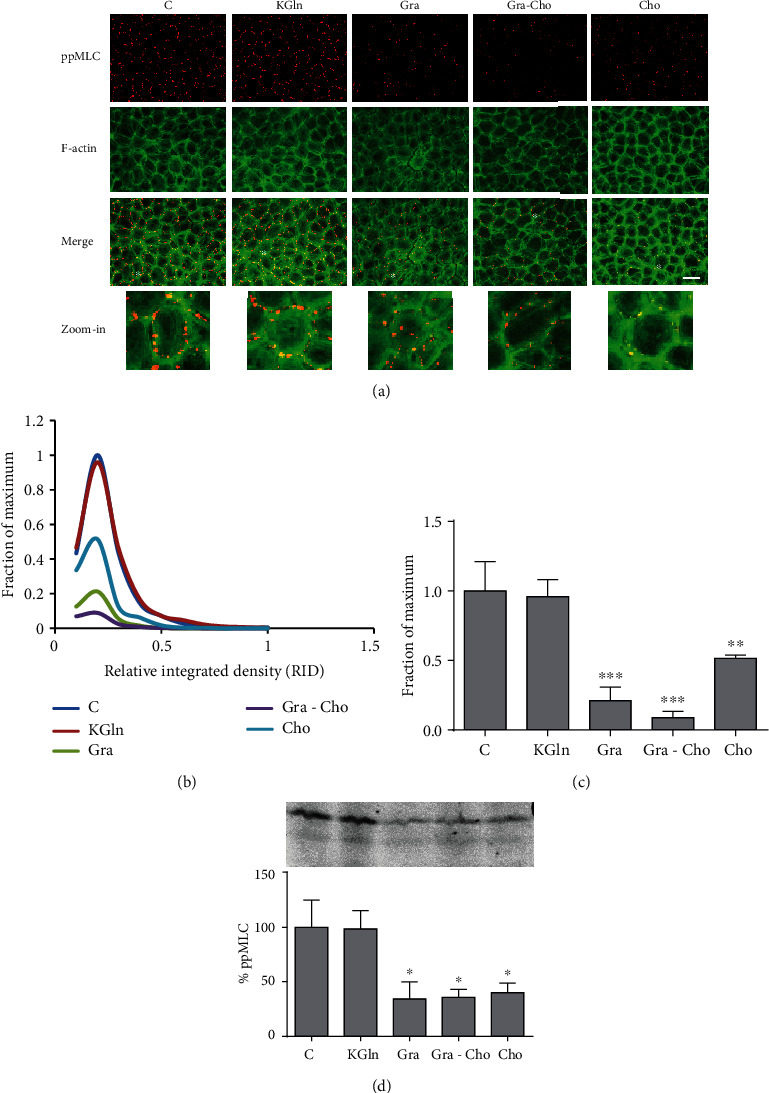
Effect of gramicidin A on MLC diphosphorylation in BCE cells. (a) Double-stained images of ppMLC and F-actin for different conditions (C: control; KGln; Gra; Gra-Cho; Cho). The asterisks in the merged images indicate cells enlarged for better visualizations (zoom-in). (b) Frequency distributions of the relative integrated densities (RID) of the dot signals with respect to control, determined from immunofluorescence images. For illustrative purposes, the distributions are represented by continuous lines connecting the corresponding values for each interval. (c) Plots of the means of the highest frequency values of IDs (corresponding to the 0.1–0.2 interval of RID values, where every condition exhibited its maximum). Asterisks indicate significant differences with respect to control (^∗∗^*p* < 0.01; ^∗∗∗^*p* < 0.0001). The signal for Cho is also significantly different with respect to Gra (*p* < 0.05) and Gra-Cho (*p* < 0.01) (not indicated in the figure). (d) Western blot of ppMLC. The plot shows the ID percentages with respect to control obtained from densitometric analysis. Asterisks indicate significant differences with respect to control (^∗^*p* < 0.05). Load and transfer controls and quantifications were done as described in Section 2.5. A representative blot is shown. Bar: 30 *μ*m.

**Figure 4:**
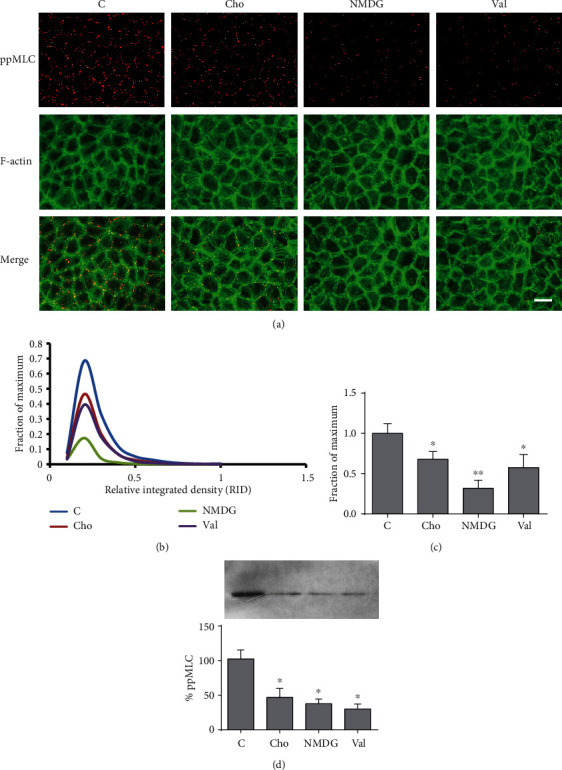
Effect of plasma membrane hyperpolarization on MLC diphosphorylation in BCE cells. (a) Double-stained images of ppMLC and F-actin for different conditions (C: control; Cho; NMDG; Val). (b) Frequency distributions of the relative integrated densities (RID) of the dot signals with respect to control, determined from immunofluorescence images. For illustrative purposes, the distributions are represented by continuous lines connecting the corresponding values for each interval. (c) Plots of the means of the highest frequency values of IDs (corresponding to the 0.1–0.2 interval of RID values, where every condition exhibited its maximum). Asterisks indicate significant differences with respect to control (^∗^*p* < 0.05; ^∗∗^*p* < 0.01). (d) Western blot of ppMLC. The plot shows the ID percentages with respect to control obtained from densitometric analysis. Asterisks indicate significant differences with respect to control (^∗^*p* < 0.05). Load and transfer controls and quantifications were done as described in Section 2.5. A representative blot is shown. Bar: 30 *μ*m.

In MDCK cells, Szászi and coworkers [6] found that PMP depolarization determines an increase in the biphosphorylated form of the myosin light chain (ppMLC). However, in BCE cells, PMP depolarization provokes a decrease in the monophosphorylated form without modifications in ppMLC [22]. As previously described [22, 27], in confluent BCE cells under control conditions, ppMLC localizes at the actomyosin peripheral ring (Figure 3(a), control). While KGln (Figure 3(a)) or other depolarizing solutions (NaGln or Na_2_SO_4_, [22]) did not, as expected, produce any effect on ppMLC, gramicidin, either as a depolarizing (i.e., in CS) or hyperpolarizing (i.e., in Cho) agent, determined a significant decrease in the amount and intensity of the ppMLC peripheral dots (Figure 3(a)). Choline by itself also decreased ppMLC, although to a lesser extent (Figure 3(a)), as well as other hyperpolarizing solutions (Figure 4). As in Section 3.1, we confirmed that the effect of choline was mediated by PMP hyperpolarization by comparison with other hyperpolarizing treatments (Figure 4). It can also be observed in the merged images (Figure 3(a)) that, by itself, Gra promotes some degree of delocalization of ppMLC, an effect that can be better appreciated in the zoom-in images. The findings revealed in Figures 3(a) and 4(a) are also evident in the corresponding histograms of the integrated density of the fluorescence signals (Figures 3(b) and 4(b)). The determination of the statistical significance of the integrated density data is presented in Figures 3(c) and 4(c). The corresponding Western blot studies are in agreement with the immunofluorescent results (Figures 3(d) and 4(d)). As with the increase in pERK, the results of this section suggest that the effect of gramicidin on ppMLC is independent of its capacity to modify the electrical conductance of the plasma membrane.

### 3.3. Effect of Gramicidin A on Tyrosine Phosphorylation

**Figure 5:**
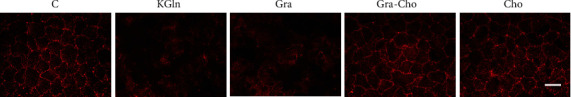
Effect of gramicidin A on tyrosine phosphorylation in BCE cells. Immunofluorescent staining of pTyr for different conditions (C: control; KGln; Gra; Gra-Cho; Cho). Bar: 30 *μ*m.

The results above reveal that gramicidin A affects the activity and localization of some signaling intermediates by itself, independently of its electrical effects as an ionophore of the plasma membrane. However, as mentioned, in previous work, we reported that incorporation of gramicidin to diverse epithelia in culture determines characteristic rearrangements of the actin cytoskeleton that indeed depend on its effects on the PMP [5, 20].  Figure 5 shows that the cellular localization of tyrosine-phosphorylated proteins is modified by gramicidin due to its ionophoric properties. Tyrosine phosphorylation is an ubiquitous posttranslational modification that participates in crucial signaling pathways of diverse cellular processes, such as cell proliferation, cell differentiation, cell adhesion, and metabolism [28]. As can be seen, both gramicidin (in depolarizing conditions, see above) and other depolarizing agents (KGln, Figure 5, and NaGln or Na_2_SO_4_, not shown) determine a displacement of the peripheral fluorescence signal of phosphotyrosine (pTyr) to the cytoplasm in some cells and loss of signal in patches of cells. When extracellular sodium is replaced by choline, gramicidin has no effect on pTyr distribution, similarly to that observed for choline alone or other hyperpolarizing agents (i.e., NMDG, not shown). These results suggest that the effect of gramicidin on the localization of the pTyr signal is due to its properties as a depolarizing agent. This finding also contributes to the concept that unspecific modifications of the PMP affect signaling cellular pathways.

## 4. Discussion

Gramicidin has been employed to modify the plasma membrane potential and to produce changes in the intracellular composition of sodium and potassium, with diverse experimental objectives. In this way, the ionophore has been utilized to obtain calibration curves for measurements of the PMP [6–8], to study the effect of PMP depolarization on several properties, such as the action of drugs [9], superoxide production [29], cytoskeletal organization [5, 20], or cell proliferation [10], and to study the effect of the modifications in the cellular concentrations of sodium and potassium on cell volume regulation [30–32] and intercellular junction organization [33]. It has been shown that the incorporation of gramicidin to the plasma membrane provokes the activation of the sodium pump, as a consequence of the increase in intracellular sodium and the concomitant decrease in potassium [34]. In turn, activation of the enzyme determines a higher rate of ATP utilization with the consequent increase in oxygen consumption [34–36]. In this way, gramicidin has also been employed to promote modifications in ATP metabolism [37], therefore also ultimately a consequence of its properties as an ionophore. The mechanisms underlying other reported effects of gramicidin have not yet been clearly established. Thus, in several tumor cell lines, gramicidin provokes cellular and mitochondrial morphological alterations, modifies the expression of specific proteins, and, in general, determines a decrease in cell viability [38–40].

In this study, we present evidence that some of the cellular effects of gramicidin cannot be interpreted in terms of its ionic or electrical properties as an ionophore of the plasma membrane. Thus, incorporation of the drug in BCE cells in culture modifies the phosphorylation level and localization of some key signaling intermediates related, among other cellular properties, to cytoskeleton organization. In this respect, gramicidin increases nuclear pERK and decreases ppMLC, both under culture conditions where the drug produces either depolarization or hyperpolarization of the PMP. Alternative depolarizing or hyperpolarizing procedures do not provoke similar responses to those to gramicidin. Thus, for the case of pERK, PMP depolarization does not modify the amount or localization of pERK, as we have also shown previously [22], whereas hyperpolarization determines an increase in pERK without augmenting its nuclear signal. However, both under depolarizing and hyperpolarizing conditions, gramicidin provokes an increase in pERK with a significant nuclear component. While PMP depolarization does not modify ppMLC (this study and [22]), hyperpolarization decreases its signal. Irrespective of the electrical condition, gramicidin provokes a strong decrease in MLC diphosphorylation. To the best of our knowledge, the present study constitutes the first report to demonstrate the existence of cellular effects of gramicidin at low concentrations, like the ones utilized here, not attributable to its properties as an ionophore capable of modifying the electrical conductance of the plasma membrane.

**Figure 6:**
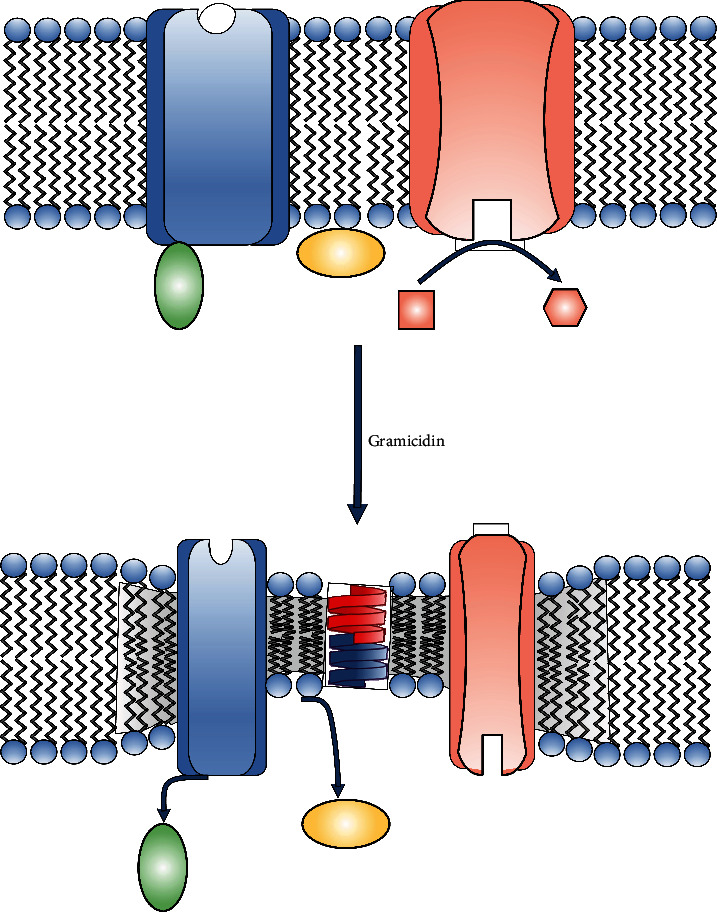
Hypothetical mechanism of the direct effects of gramicidin. Besides determining modifications in the membrane conductance, incorporation of gramicidin to a lipid membrane provokes alterations in its lipid environment (represented as gray background in the figure). When the ionophore adopts its dimeric form (red and dark blue monomers), it determines a change in the neighboring lipid composition, with a predominance of short-chain phospholipids. In addition, it provokes a compression of medium- and long-chain phospholipids. As a consequence, the bilayer thickness of the lipid ambient close to the channel decreases and there is a shift in mechanical stress. These alterations could affect the activity of integral membrane proteins in the proximity of the channel, either enzymes (pink) or receptors (blue), in turn associated with other binding proteins (green). Peripheral membrane proteins, more loosely associated with the membrane (yellow), could also be affected.

What could be the mechanism through which gramicidin provokes cellular effects independently of its properties as an ionophore? Incorporation of gramicidin into membranes determines modifications in the composition and degree of compression of the lipids adjacent to the channel, ultimately decreasing the bilayer thickness, particularly when it adopts its dimeric, conductive form [14, 15, 41, 42]. A plausible hypothesis for the mechanism underlying the effects communicated in this study could involve these properties of gramicidin. Based upon this, in Figure 6, we present a scheme of the possible events that could account for the results of this work. The alterations provoked by gramicidin on its immediate lipid environment could affect the conformation of neighboring membrane proteins, both integral and peripheral, and hence their activity. For the case of ERK, one or more membrane receptors involved in its regulatory pathways could be affected by the changes in the lipid environment promoted by gramicidin. In this respect, both ERK activation and inhibition can be triggered by membrane protein receptors [43–45]. Similarly, in this line of thought, the decrease in the level of MLC phosphorylation, provoked as a direct effect of gramicidin, could result from modifications in the activity of a protein receptor and/or enzyme of the plasma membrane. Different pathways triggered by stimuli at the plasma membrane level participate in the regulation of MLC phosphorylation [46–48]. For instance, activation of the transmembrane adenylyl cyclase could represent a candidate to account for the effects of gramicidin reported in this study. The resulting increase in cAMP would in turn activate PKA, a well-known positive modulator of the MLC phosphatase, determining a decrease in ppMLC [49].

Another finding of this work is that nonspecific modifications of the PMP elicit different responses in signaling intermediates in BCE cells. In this way, hyperpolarization determines an increase in the pERK signal at the cytoplasm and a decrease of ppMLC at the cell periphery. In turn, depolarization does not affect pERK or ppMLC (see also [22]) but decreases tyrosine phosphorylation (this study), both at the cell periphery and the cytoplasm. These results contrast with those by Szászi and coworkers [6, 50] for MDCK and other kidney cell lines, where plasma membrane depolarization increases the phosphorylation levels of ERK1/2 and MLC. This suggests that the effects of PMP modifications are tissue-specific. The studies on the role of plasma membrane potential on signaling pathways of nonexcitable cells have received renewed interest in the last years, since diverse works have shown an important contribution of bioelectrical phenomena on cell and organ development and cancer progression [51–56]. In view of the frequent employment of gramicidin to experimentally manipulate the plasma membrane potential, the present study contributes with a note of caution on the interpretation of its effects.

## 5. Conclusions

In this study, we have provided evidence that gramicidin A modifies the activity of signaling pathways in a manner independent of its properties as a cationic channel of the plasma membrane. This finding could be useful for those authors that employ gramicidin to experimentally manipulate the plasma membrane potential, in order to prevent misinterpretations of the cellular effects of the drug.

## Data Availability

Data that support the findings of this study are available from the authors upon reasonable request.
